# Social determinants of financial stress and association with psychological distress among young adults 18–26 years in the United States

**DOI:** 10.3389/fpubh.2024.1485513

**Published:** 2025-01-07

**Authors:** Anaiya Nasir, Umair Javed, Kobina Hagan, Ryan Chang, Harun Kundi, Zahir Amin, Sara Butt, Sadeer Al-Kindi, Zulqarnain Javed

**Affiliations:** ^1^St. Agnes Academy, Houston, TX, United States; ^2^Combined Military Hospital Medical Center, University of Health Sciences, Lahore, Pakistan; ^3^Houston Methodist DeBakey Heart and Vascular Center, Houston, TX, United States; ^4^Baylor College of Medicine, Houston, TX, United States; ^5^Cardiovascular Research Foundation, New York, NY, United States; ^6^University of Texas Medical Branch, Galveston, TX, United States; ^7^Center for Health Data Science and Analytics, Houston Methodist, Houston, TX, United States; ^8^Department of Cardiology, Houston Methodist DeBakey Heart and Vascular Center, Houston, TX, United States; ^9^Center for Cardiovascular Computational Health & Precision Medicine (C3-PH), Houston Methodist, Houston, TX, United States

**Keywords:** financial stress, mental health, wellbeing, young adults, social determinants of health

## Abstract

**Introduction:**

Financial stress (FS) during young adulthood may have lasting effects on financial security, physical health, and overall wellbeing. This study examines the burden, social determinants and mental health consequences of experienced FS among young adults in the United States, based on objective measures of financial stress.

**Methods:**

We studied young adults aged 18–26 years using pooled data from the 2013–18 National Health Interview Survey. FS was assessed as an aggregate score (6–24) based on worry about six life tasks: paying for: monthly bills, housing expenses, healthcare, illness/accident, maintaining standard of living, saving money for retirement. Individuals in the highest quartile of the score were defined as having high FS. Psychological distress (PD) was measured using the six-item Kessler Psychological Distress Scale (K6); high PD was defined as total K6 score ≥13. Multivariable ordinal and logistic regression models were used to assess key social determinants of FS and the association between FS and PD, respectively.

**Results:**

Study sample included 19,821 individuals aged 18–26 years (34 million annualized). Overall, 17% (5.8 million nationally) of young adults reported high FS. Female, non-Hispanic Black and Hispanic, low income/low education, uninsured, non-citizen and comorbidities were associated with high FS burden. In fully adjusted models, high FS was associated with over 6-fold (OR = 6.17, 95% CI 4.43–8.61) higher risk of high PD.

**Discussion:**

One in six young adults in the US experiences high FS, which portends high risk of PD. These findings should inform stakeholder deliberations to identify and mitigate the unintended mental health consequences of FS in this vulnerable population.

## Introduction

Young adulthood, typically spanning ages 18–26, signifies a pivotal transition period in an individual's life, significantly influencing long-term economic stability and physical and mental health (1). This age group has unique financial needs, influenced by evolving social and environmental circumstances which may have varying effects on psychological and emotional wellbeing (1, 2). While nearly 30% of US adults report experiencing financial instability overall, relatively little is known about the burden, social determinants and mental health sequelae of financial stress (FS) specifically in the young adult population (3). Despite the critical importance of young adulthood toward growth and development over the life course, this population remains relatively understudied compared to other life stages (2).

Financial stress (FS), which is traditionally defined as an emotional response to economic hardship, is well-recognized for its profound effects across various life domains during later years, yet its impact on young adults remains relatively understudied (3, 4). It has also been defined as a subjective phenomenon, where an adverse financial situation can lead to varying degrees of perceived stress, resulting in mental, physical, and emotional distress (7). As adolescents transition into young adulthood and navigate newfound independence, they encounter a multitude of financial responsibilities including education expenses, rent, and future financial planning (5). The consequences of FS during this crucial developmental phase may extend beyond mere economic concerns, significantly impacting long-term physical and mental health, quality of life, and coping abilities (6). Furthermore, psychological distress (PD), encompassing symptoms such as anxiety, depression, and stress may be exacerbated by FS, creating a cycle whereby economic hardships intensify mental health struggles with short and long-term health consequences (3–5).

Understanding the burden and key social drivers of FS, as well downstream effects on PD among young adults is paramount for creating targeted interventions and policy initiatives to mitigate economic hardships and promote mental health assistance. Moreover, identifying vulnerable groups and addressing modifiable clinical and social determinants of health (SDOH) is crucial for tailored interventions to address the unique needs of young adults and alleviate the potential adverse consequences of FS on their health and overall wellbeing.

The objective of this study is to comprehensively investigate the intersection of FS with sociodemographic factors and its potential impact on mental health among young adults aged 18–26 years in the United States (US). Structural disparities, as noted within differences in access to education, access to health care, and stable employment, are some of the major challenges that young adults will face during this transitional period in life. These challenges compounded with uncertain income and housing circumstances can further possible financial stress and can probably result in negative psychological impacts (2–5). By examining the complex interactions among social inequities, individual level financial distress burden, and psychological distress this study aims to contribute to a deeper understanding of the challenges faced by young adults that can inform future evidence-based interventions to promote their overall wellbeing.

## Methods

### Data source and study sample

We conducted a cross-sectional analysis utilizing data from the National Health Interview Survey (NHIS) spanning from 2013 to 2018. This dataset, collected annually through complex, multi-stage sampling techniques, provides comprehensive insights into the non-institutionalized US population. Our study focused on young adults aged between 18 and 26 years. The data was extracted from the Sample Adult Core files, augmented with demographic and socioeconomic indicators, health status metrics, healthcare service utilization, and health-related behavioral data of the US adult population. As NHIS data is publicly available in a de-identified format, our study was exempt from Institutional Review Board oversight.

### Study outcomes

#### Financial stress (FS)

FS was assessed based on six questions regarding concerns about various financial matters, including monthly bills, rent/mortgage and housing expenses, routine healthcare expenses, costs associated with illness or accidents, maintaining a standard of living, and saving for retirement, as shown in Supplementary Table S1. Responses to these questions were captured on an ordinal scale ranging from “Not Worried at All (1),” “Not Too Worried (2),” “Moderately Worried (3),” to “Very Worried (4).” We aggregated the responses into an index score ranging from 6 to 24, with higher scores indicating greater FS. The final score was categorized with quartiles (Qx), with Q1-4 corresponding to no FS, low, moderate, and high FS, respectively, as done previously (8, 9).

#### Psychological distress

We utilized the six-item Kessler Psychological Distress Scale (K6) to identify individuals experiencing psychological distress over a 30-day recall period. The K6 queries respondents on the frequency with which respondents felt sadness, nervousness, restlessness, hopelessness, perceived effort in daily tasks, and worthlessness: none of the time (0); little of the time (1); some of the time (2); most of the time (3); all of the time (4). Responses were recorded on a 6-point Likert scale, with summed scores ranging from 0 to 24. Individuals scoring 13 or higher were classified as having severe psychological distress as previously described (10, 11).

#### Covariates

Various covariates were included in our analysis, encompassing demographic factors such as sex, race/ethnicity, family income, marital status, insurance status, education level, immigration status, region of residence, and the number of chronic comorbidities. Chronic comorbidities were self-reported and included emphysema, chronic obstructive pulmonary disease, asthma, gastrointestinal ulcer, cancer (any), arthritis (including arthritis, gout, fibromyalgia, rheumatoid arthritis, and systemic lupus erythematosus), any kind of liver condition, and “weak/failing” kidneys. These were defined as binary variables (0/1) and were subsequently aggregated and categorized into comorbidity index: 0 or ≥1 comorbidities.

### Statistical analysis

Participant descriptive characteristics were presented as unweighted counts and means with accompanying weighted proportions and 95% confidence intervals (CI), overall and across FS categories. Age was described as both a continuous (mean, SD) and categorical variable. The burden of FS was reported across various social, demographic and clinical subgroups. Chi-squared tests were used to assess differences in social, demographic and clinical determinants by FS status.

We examined the associations between individual sociodemographic characteristics and FS using multivariable survey-specific ordinal logistic regression models to report odds ratios (OR) and 95% CI for high financial stress (vs. combined no/low/moderate stress), adjusted for sociodemographic and clinical risk factors.

Additionally, we assessed the proportion of young adults reporting psychological distress, both overall and stratified by the presence of high FS. Finally, we explored the association between FS and psychological distress using unadjusted and multivariable-adjusted, survey-specific logistic regression models.

## Results

This study encompassed a robust sample of 19,821 young adults aged between 18 and 26 years, drawn from the National Health Interview Survey (NHIS) spanning the years 2013–2018, corresponding to ~34 million individuals annually. The mean age of participants was 22.4 (SD = 2.5) years, with a nearly equal gender distribution. Overall, 39% of participants identified as non-Hispanic Black or Hispanic and 10% reported immigrant or naturalized citizen status. A total of 42%−44% reported less than a high school education and belonging to a low-income group, respectively. Additionally, 18% reported being uninsured, while 5% reported having ≥1 comorbid conditions. No, low, moderate, and high financial stress, respectively, corresponded to index scores of 6–7, 8–12, 13–16, and 17–24, and represented an annualized population of 10.3, 11.6, 6.2, and 5.8 million young adults, respectively. Most marginalized groups among young adults reported a higher burden of FS, with a substantially higher prevalence in minority racial/ethnic subgroups and those with low income and education (Table 1).

**Table 1:** Descriptive characteristics for adults 18–26 years by financial stress status, from the National Health Interview Survey 2013–18.

	Total^*^	**No FS**	**Low FS**	**Moderate FS**	**High FS**
No. (*N*)	19,821	5,772	6,874	3,758	3,417
Estimated US population (weighted %)	33,889,626 (100)	10,311,531 (30.4)	11,603,681 (34.2)	6,209,287 (18.3)	5,765,127 (17.0)
Age, mean (SD)	22.4 (2.5)	21.6 (2.6)	22.4 (2.5)	22.8 (2.4)	23.1 (2.4)
**Sex**, ***n*** **(weighted %)**					
Male	9,497 (50.6)	3,033 (32.9)	3,366 (34.0)	1,671 (17.4)	1,427 (15.6)
Female	10,324 (49.4)	2,739 (27.9)	3,508 (34.5)	2,087 (19.3)	1,990 (18.4)
**Race/ethnicity**, ***n*** **(weighted %)**					
Non-Hispanic white	12,398 (61.3)	4,020 (33.4)	4,532 (35.6)	2,289 (18.1)	1,557 (12.9)
Non-Hispanic Black	3,001 (15.7)	804 (28.7)	945 (31.8)	547 (17.2)	705 (22.3)
Hispanic	4,422 (23.0)	948 (23.6)	1,397 (32.3)	922 (19.8)	1,155 (24.3)
**Family income**, ***n*** **(weighted %)**					
Middle/high-income	8,644 (56.2)	2,680 (32.7)	3,261 (37)	1,569 (17.2)	1,134 (13.1)
Low-income	10,272 (43.8)	2,777 (26.8)	3,325 (31)	2,044 (20.1)	2,126 (22)
**Education**, ***n*** **(weighted %)**					
≥Some college	12,286 (58.4)	3,667 (30)	4,565 (37.2)	2,353 (18.5)	1,701 (14.4)
≤ High school	7,535 (41.6)	2,105 (31)	2,309 (30.1)	1,405 (18.1)	1,716 (20.7)
**Citizenship**, ***n*** **(weighted %)**					
US citizens	17,874 (90.3)	5,386 (31.4)	6,277 (34.6)	3,388 (18.3)	2,823 (15.7)
Naturalized citizens	597 (3.3)	131 (25.5)	241 (40.1)	112 (17.8)	113 (16.6)
Immigrant non-US citizens	1,329 (6.4)	251 (20.4)	348 (25.8)	254 (18.6)	476 (35.2)
**Insurance status**, ***n*** **(weighted %)**					
Insured	16,253 (82.5)	5,267 (33.5)	5,940 (36)	2,951 (17.4)	2,095 (13.1)
Uninsured	3,568 (17.5)	505 (15.9)	934 (25.9)	807 (22.7)	1,322 (35.5)
**Region**, ***n*** **(weighted %)**					
Northeast	2,740 (15.5)	868 (33.3)	927 (33)	500 (17.1)	445 (16.6)
Midwest	4,787 (24.4)	1,477 (30.7)	1,690 (35.6)	932 (19.4)	688 (14.3)
South	7,158 (37.0)	2,071 (31.1)	2,448 (33.6)	1,300 (17.3)	1,339 (18.1)
West	5,136 (23.1)	1,356 (27.2)	1,809 (34.8)	1,026 (19.7)	945 (18.4)
**Marital status**, ***n*** **(weighted %)**					
Not married	19,443 (98.8)	5,705 (30.5)	6,772 (34.4)	3,661 (18.2)	3,305 (16.8)
Currently/previously married	361 (1.22)	61 (19.6)	99 (23.9)	94 (26.1)	107 (30.5)
**Psychological distress**, ***n*** **(weighted %)**					
No	19,073 (96.8)	5,658 (30.9)	6,733 (34.9)	3,593 (18.2)	3,089 (16.1)
Yes	645 (3.2)	79 (14.6)	107 (16.2)	146 (24)	313 (45.2)
**Comorbidities**, ***n*** **(weighted %)**					
None	18,862 (95.16)	5,658 (30.8)	6,600 (34.5)	3,521 (18.1)	3,163 (16.6)
Any	959 (4.84)	86 (23)	274 (27.9)	237 (22.9)	254 (26.2)

^*^All *p*-values are < 0.001.

In our study, 3.3% of young adults reported experiencing PD. Prevalence of PD increased substantially with increasing FS burden, from 15% for those reporting no financial worry to over 45% for those reporting severe financial stress (Table 1).

**Figure 1:**
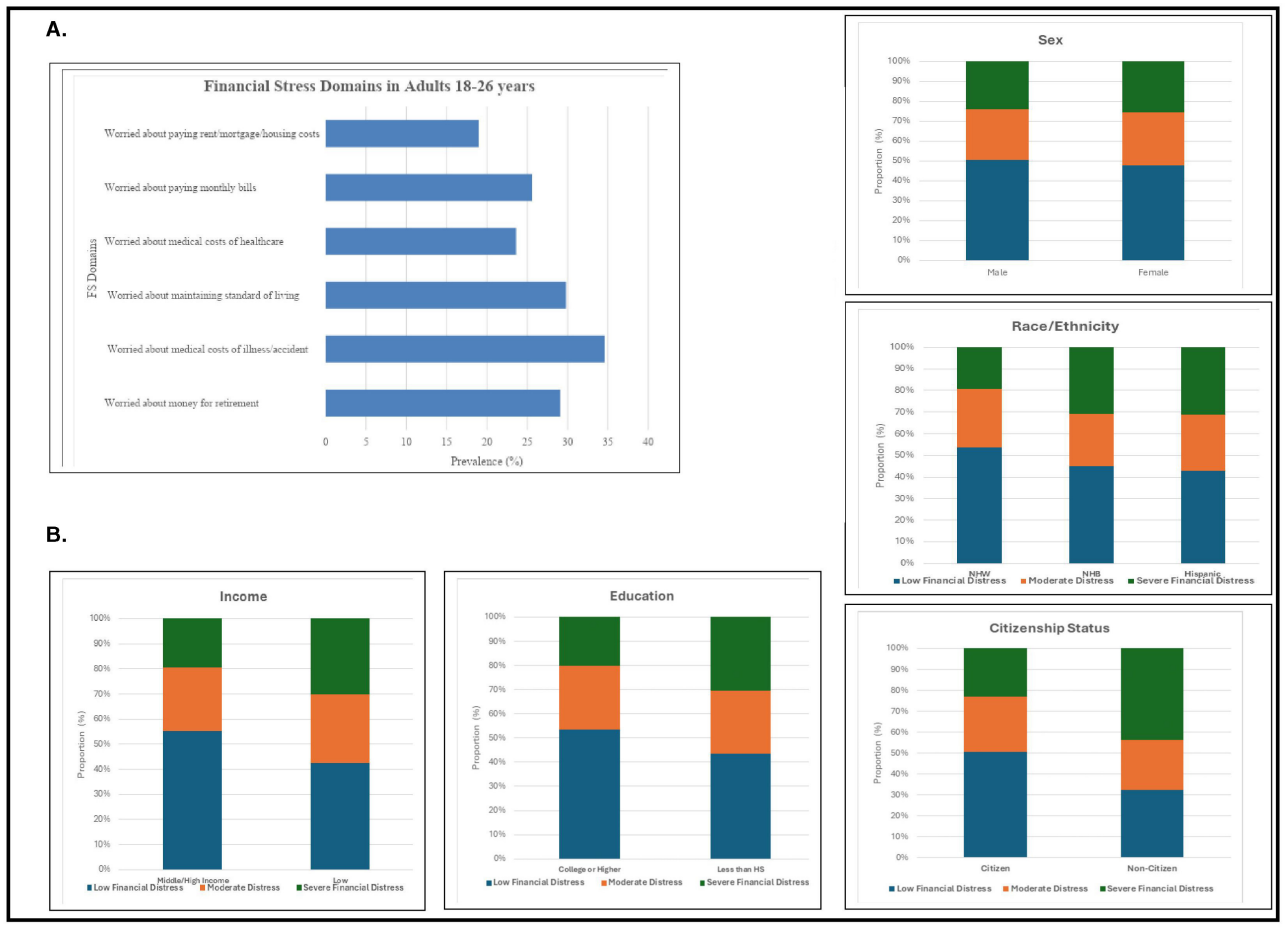
**(A)** Burden of financial stress by individual domains. **(B)** Financial stress prevalence by sociodemographic subgroups.

The survey delved into various domains of FS among young adults. Notably, medical costs associated with illness or accidents emerged as a significant source of anxiety, with 34.6% of respondents expressing worry (combining those who were very and moderately worried). This was followed by concerns about maintaining the standard of living (29.8%) and having sufficient retirement savings (29.1%). Worries related to medical costs of healthcare were also notable, with 23.6% expressing concern, whereas 19% of respondents expressed worry about paying monthly bills and rent/mortgage/housing costs. Sociodemographic subgroup analysis revealed that minoritized racial/ethnic subgroups (non-Hispanic Black, Hispanic), non-citizens and those with low income and low education experienced a higher burden of FS relative to their counterparts (Figures 1A, B).

**Figure 2:**
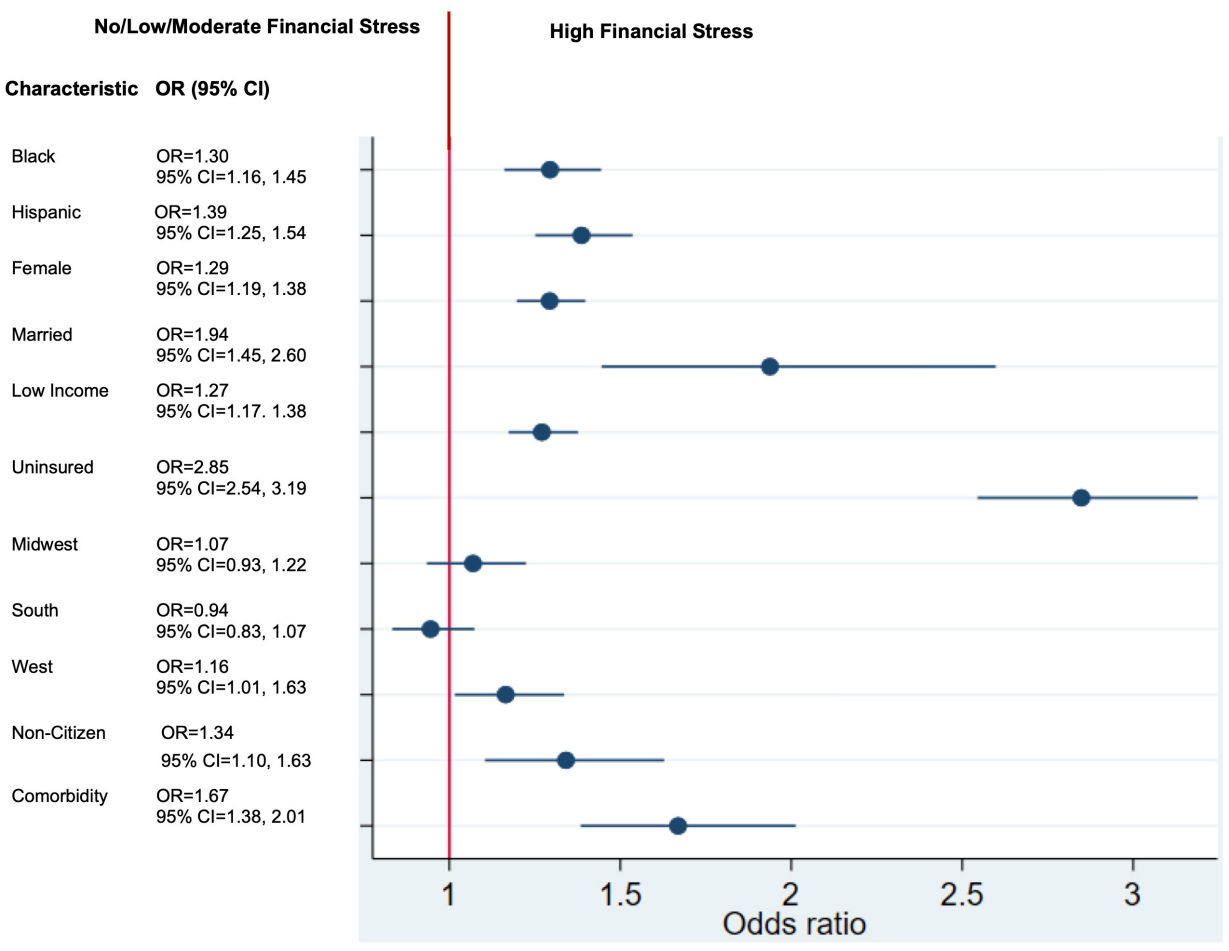
Social determinants of financial stress.

In multivariate analyses, females exhibited a 29% higher likelihood of experiencing FS compared to males (OR: 1.29, 95% CI: 1.19–1.38). Racial and ethnic minorities also demonstrated elevated odds of FS, with non-Hispanic Black individuals experiencing a 30% higher likelihood (OR: 1.30, 95% CI: 1.16–1.45) and Hispanic individuals having a nearly 40% higher likelihood (OR: 1.39, 95% CI: 1.25–1.54) compared to non-Hispanic White individuals. Individuals who reported being currently or previously married had nearly 50% higher odds of FS compared to those who never married (OR: 1.94, 95% CI: 1.45–2.60). Low-income (OR: 1.27, 95% CI: 1.17–1.38) and immigrant/non-citizen individuals (OR: 1.34; 95% CI: 1.10–1.63) were also at an increased risk of FS, with nearly 30% higher likelihood compared to their middle/high-income and citizen counterparts, respectively. Uninsured status was associated with ~3-fold increased odds of FS (OR: 2.85, 95% CI: 2.54–3.19). Additionally, individuals with comorbidities demonstrated a 70% higher likelihood of financial stress compared to those without comorbidities (OR: 1.67, 95% CI: 1.38–2.01) (Figure 2).

We found a strong multivariable association between severity of FS and PD. Individuals facing high FS exhibited a 6-fold increase in the likelihood of experiencing PD (OR = 6.17; 95% CI: 4.43–8.61). Importantly, these associations remained robust even after adjusting for social, demographic, and disease burden factors (Table 2).

**Table 2:** Financial distress and psychological distress: multivariable regression, from the National Health Interview Survey 2013–18.

		**OR for psychological distress**		
		**OR (95% CI)**	**OR (95% CI)**	**OR (95% CI)**
		**Model 1**	**Model 2**	**Model 3**
Financial stress	No financial stress	Reference	Reference	Reference
	Low financial stress	0.98 (0.64, 1.51)	1.05 (0.68, 1.62)	1.04 (0.68, 1.62)
	Moderate financial stress	2.81 (1.95, 4.04)	2.86 (1.97, 4.15)	2.80 (1.92, 4.07)
	High financial stress	6.30 (4.59, 8.64)	6.44 (4.63, 8.96)	6.17 (4.43, 8.61)

Model 1: Adjusted for sex, race/ethnicity.

Model 2: Adjusted for sex, race/ethnicity, marital status, income status, education, insurance status, region, and citizenship status.

Model 3: Adjusted for sex, race/ethnicity, marital status, income status, education, insurance status, region, citizenship status, and comorbidity status.

## Discussion

In this nationally representative study, we observed that nearly 17% (estimated 5.8 million individuals) of young adults aged 18–26 reported high FS. Notably, marginalized groups, including racial and ethnic minorities, young women, individuals from low-income backgrounds, those with lower education levels, uninsured, married, immigrant non-citizens, and those with higher burden of underlying diseases had disproportionately higher rates of FS. Our study revealed a strong, consistent association between high FS and PD among young adults. Specifically, individuals experiencing high FS were 6-fold more likely to report severe PD compared to their counterparts, with 8.4% of young adults with high FS reporting PD compared to 1.6% with low FS.

While our findings corroborate prior literature on the sociodemographic disparities in economic strain in US adults, our study addresses several key knowledge gaps regarding health and wellbeing during emerging adulthood as a critical transition phase in life. Ours is the first population-based US study to (i) describe the burden of FS, (ii) identify the key social, demographic and clinical determinants of FS, (iii) describe socio-demographic heterogeneity in FS burden, and (iv) examine the association between FS and PD in a nationally representative sample of young adults 18–26 years of age. With a sample size of ~20,000 individuals, representing nearly 34 million young adults nationwide, our findings are generalizable to young adults throughout the US. Our findings call for greater attention to addressing the burden and long-term sequelae of financial strain in this vulnerable population.

Overall, 1 in every 6 young adults reported a high burden of FS. While literature has suggested a higher financial burden in the general adult population, there are specific facets that impact the younger population more profoundly. We found that the most frequent financial worry in this group was the cost for serious medical events (31%), followed by maintaining standard of living (27%) which is in contrast to the challenges experienced by the general adult population such as paying for their children's academic tuition and retirement concerns, as reported previously (12). Moreover, stress in the young adult population has broader societal implications given its relationship with job strain (13). This is particularly relevant in young adults who are in early career facing higher expectations despite low experience (14) and report feeling greater work related stress (15). This further leads to negative impacts on mental health in previously healthy young adults (16, 17). Mental health is a key determinant of job performance and an established risk factor for premature exit from paid employment (18). Given the long-term individual and societal implications of health and wellness in young adulthood, our study underscores the urgency of informed policy decisions and supportive environments to mitigate financial strain and empower young adults to thrive and achieve their full potential.

Our assessment of the FS-PD association is based on robust statistical modeling, accounting for traditional and non-traditional risk factors including SDOH, demographics and established clinical determinants. In general, prior observational studies in this age group do not adequately account for the multitude of social determinants that differentially impact this age group. Our findings reveal that young women, racial and ethnic minorities, individuals with lower education levels, those from low-income households, the uninsured, married, or divorced individuals, non-citizen immigrants, and those with a higher underlying disease burden are disproportionately affected by high FS.

It is well-established that young women often experience gender pay gaps and are further disadvantaged with caregiving responsibilities, placing them at a higher risk of financial strain compared to men (19). Women may also be at a higher risk of experiencing PD due to underlying economic instability relative to their counterparts (12). Prior studies have also highlighted how racial and ethnic minorities face systemic barriers in accessing quality educational and employment opportunities that can contribute to their economic vulnerability during young adulthood (20). Similarly, those with lower levels of education may never have the opportunity to develop the necessary skills and qualifications for well-paying jobs, perpetuating financial instability (21). Additionally, young adults from low-income households may struggle to meet their basic living expenses, which is likely to further exacerbate their susceptibility to financial stress (21). Among non-citizen immigrants, there are additional legal and socioeconomic obstacles intensifying their economic vulnerability during young adulthood (22). Similarly, uninsured patients face the financial burden of medical expenses with even routine healthcare visits (23) which may be significantly higher if faced with a severe underlying disease burden (24). These insights not only call for further research to identify the specific pathways underlying heightened risk of financial stress, but also underscore the importance for policymakers and healthcare professionals to address the structural inequalities contributing to the economic vulnerability of marginalized young adults. Ultimately, these efforts are crucial for creating more inclusive and equitable opportunities for this population.

Finally, our study shows substantial variation in burden of PD by FS severity, with over 6-fold higher odds of PD among those experiencing the highest level of financial strain, relative to those with the lowest FS. While it is well known that marginalized groups are more likely to experience PD, our findings were robust to adjustment for sociodemographic and clinical risk factors. This suggests that FS may serve as a significant independent determinant of psychological distress among young adults. Our findings underscore the critical importance of screening FS among young adults as a public health priority. Future studies should explore whether implementing screening for FS and subsequent mitigation strategies within healthcare settings could favorably impact the burden of psychological and mental distress among younger individuals.

Our study has a few limitations. Given the cross-sectional nature of the data, we were unable to determine if FS had longitudinal impacts on mental health, and if feelings of FS were permanent, therefore limiting our ability to infer directional relationships between social risks, financial stress, and psychological distress. Secondly, the FS questionnaire utilized in this study may not have captured all relevant sources of financial worry, particularly those more pertinent to young adults, such as childcare and food expenses. This may have underestimated the true extent of financial distress experienced by this population. Thirdly, NHIS data does not include institutionalized and homeless populations, potentially leading to an underestimation of the overall burden of financial distress, especially considering the heightened vulnerability of these groups. Finally, the reliance on self-reported survey data introduces the possibility of recall bias, wherein participants may inaccurately recall or misrepresent their experiences with financial stress and psychological distress.

In conclusion, our study highlights the pervasive nature of financial stress among young adults, with a notable 17% reporting significant financial distress, translating to ~5.8 million young adults in the United States alone. This burden is further compounded by a 6-fold increase in the risk of PD among those experiencing financial strain. Notably, marginalized sociodemographic groups bear a disproportionate burden of FS, highlighting systemic inequities in economic wellbeing. There is a pressing need to develop comprehensive national health research and policy agenda tailored specifically for young adults. Such initiatives should prioritize the identification, intervention, and mitigation of the unintended consequences of FS in this emerging adulthood life period, with particular attention to addressing disparities among socially marginalized communities.

## Data Availability

The datasets presented in this study can be found in online repositories. The names of the repository/repositories and accession number(s) can be found at: https://www.cdc.gov/nchs/nhis/nhis_2013_data_release.htm.

## References

[1] Halfon N Forrest CB Lerner RM Faustman EM editors. Handbook of Life Course Health Development [Internet]. Cham, CH: Springer (2018). 10.1007/978-3-319-47143-3 31314220

[2] National Research Council Institute Institute of Medicine Board Board on Children Youth and Families Committee Committee on Improving the Health Safety and and Well-Being of Young Adults. Investing in the Health and Well-Being of Young Adults. Washington, DC: National Academies Press (2015).

[3] Ryu S Fan L. The relationship between financial worries and psychological distress among US. Adults J Fam Econ Issues. (2023) 44:16–33. 10.1007/s10834-022-09820-9 35125855 PMC8806009

[4] Lin P Hillstrom K Gottesman K Jia Y Kuo T Robles B. Financial and other life stressors, psychological distress, and food and beverage consumption among students attending a large California State University during the COVID-19 pandemic. Int J Environ Res Public Health. (2023) 20:3668. 10.3390/ijerph20043668 36834363 PMC9965632

[5] Guan N Guariglia A Moore P Xu F Al-Janabi H. Financial stress and depression in adults: a systematic review. PLoS ONE. (2022) 17:e0264041. 10.1371/journal.pone.0264041 35192652 PMC8863240

[6] Anthony M Sabri MF Wijekoon R Rahim HA Abdullah H Othman MA. The influence of financial socialization, financial behavior, locus of control and financial stress on young adults' financial vulnerability. Int J Acad Res Business Soc Sci. (2021) 11:289–309. 10.6007/IJARBSS/v11-i19/11738

[7] O'Neill B Sorhaindo B Prawitz A Kim J Garman ET. Financial distress: definition, effects and measurement. Consumer Interests Annu. (2006) 52:489–96.

[8] Caraballo C Valero-Elizondo J Khera R Mahajan S Grandhi GR Virani SS. Burden and consequences of financial hardship from medical bills among nonelderly adults with diabetes mellitus in the United States. Circ Cardiovasc Qual Outcomes. (2020) 13:e006139. 10.1161/CIRCOUTCOMES.119.006139 32069093

[9] Grandhi GR Valero-Elizondo J Mszar R Brandt EJ Annapureddy A Khera R. Association of cardiovascular risk factor profile and financial hardship from medical bills among non-elderly adults in the United States. Am J Prev Cardiol. (2020) 2:100034. 10.1016/j.ajpc.2020.100034 34327457 PMC8315456

[10] Kessler RC Barker PR Colpe LJ Epstein JF Gfroerer JC Hiripi E. Screening for serious mental illness in the general population. Arch Gen Psychiatry. (2003) 60:184–9. 10.1001/archpsyc.60.2.184 12578436

[11] Veldhuizen S Cairney J Kurdyak P Streiner DL. The sensitivity of the K6 as a screen for any disorder in community mental health surveys: a cautionary note. Can J Psychiatry. (2007) 52:256–9. 10.1177/070674370705200408 17500307

[12] Weissman J Russell D. Mann JJ. Sociodemographic characteristics, financial worries and serious psychological distress in US adults. Community Ment Health J. (2020) 56:606–13. 10.1007/s10597-019-00519-0 31894440

[13] Van Schaaijk A Noor Baloch A Thomée S Frings-Dresen M Hagberg M Nieuwenhuijsen K. Mediating factors for the relationship between stress and work ability over time in young adults. Int J Environ Res Public Health. (2020) 17:2530. 10.3390/ijerph17072530 32272748 PMC7177359

[14] Ilmarinen J. Work ability–a comprehensive concept for occupational health research and prevention. Scand J Work Environ Health. (2009) 35:1–5. 10.5271/sjweh.1304 19277432

[15] Hsu HC. Age differences in work stress, exhaustion, well-being, and related factors from an ecological perspective. Int J Environ Res Public Health. (2018) 16:10050. 10.3390/ijerph16010050 30585250 PMC6338997

[16] Melchior M Caspi A Milne BJ Danese A Poulton R Moffitt TE. Work stress precipitates depression and anxiety in young, working women and men. Psychol Med. (2007) 37:1119–29. 10.1017/S0033291707000414 17407618 PMC2062493

[17] Law PCF Too LS Butterworth P Witt K Reavley N Milner AJ. systematic review on the effect of work-related stressors on mental health of young workers. Int Arch Occup Environ Health. (2020) 93:611–22. 10.1007/s00420-020-01516-7 31932956

[18] Lallukka T Kronholm E Pekkala J Jäppinen S Blomgren J Pietiläinen O. Work participation trajectories among 1,098,748 Finns: reasons for premature labour market exit and the incidence of sickness absence due to mental disorders and musculoskeletal diseases. BMC Public Health. (2019) 19:1418. 10.1186/s12889-019-7753-6 31666045 PMC6821029

[19] Blau FD Kahn LM. The gender wage gap: extent, trends, and explanations. J Econ Lit. (2017) 55:789–865. 10.1257/jel.20160995

[20] Hardaway CR McLoyd VC. Escaping poverty and securing middle class status: how race and socioeconomic status shape mobility prospects for African Americans during the transition to adulthood. J Youth Adolesc. (2009) 38:242–56. 10.1007/s10964-008-9354-z 19636721 PMC4108157

[21] Luotonen N Puttonen V Rantapuska E. Ability, educational attainment, and household financial distress. J Consumer Policy. (2022) 45:655–72. 10.1007/s10603-022-09528-122563703

[22] Arega M Linggonegoro DW Dee EC Torous J. Financial worry and psychological distress among immigrants in the United States, 2013-2018. J Psychiatr Pract. (2022) 28:117–29. 10.1097/PRA.0000000000000612 35238823

[23] Jones SMW Chennupati S Nguyen T Fedorenko C Ramsey SD. Comorbidity is associated with higher risk of financial burden in medicare beneficiaries with cancer but not heart disease or diabetes. Medicine. (2019) 98:e14004. 10.1097/MD.0000000000014004 30608446 PMC6344147

[24] McKenna RM Pintor JK Ali MM. Insurance-based disparities in access, utilization, and financial strain for adults with psychological distress. Health Aff. (2019) 38:826–34. 10.1377/hlthaff.2018.05237 31059361

