# Supplementary material for: Social determinants of financial stress and association with psychological distress among young adults 18–26 years in the United States
Source: Front Public Health. 2025 Jan 7;12:1485513. doi: 10.3389/fpubh.2024.1485513 (PMC11752891; doi:10.3389/fpubh.2024.1485513)
Supplement: Supplementary file 1 [file Table_1.docx]

**SUPPLEMENT**

| **Table S1. Individual Financial Stress Items for Adults 18-26 years, from the National Health Interview Survey 2013-18.** | |
| --- | --- |
| **No. (N)** | **19,821** |
| **Estimated US Population, (weighted %)** | **33,889,626 (100)** |
|  |  |
| How worried are you about money for retirement, n (weighted %) |  |
| 1 Very worried | 2,222 (11.0) |
| 2 Moderately worried | 3,707 (18.1) |
| 3 Not too worried | 5,625 (27.5) |
| 4 Not worried at all | 8,267 (43.4) |
| How worried are you about medical costs of illness/accident, n (weighted %) | |
| 1 Very worried | 2,990 (14.4) |
| 2 Moderately worried | 4,025 (20.2) |
| 3 Not too worried | 4,944 (24.7) |
| 4 Not worried at all | 7,862 (40.7) |
| How worried are you about maintaining standard of living, n (weighted %) | |
| 1 Very worried | 1,936 (9.9) |
| 2 Moderately worried | 3,928 (19.9) |
| 3 Not too worried | 5,986 (29.2) |
| 4 Not worried at all | 7,971 (41.0) |
| How worried are you about medical costs of healthcare, n (weighted %) | |
| 1 Very worried | 1,782 (8.9) |
| 2 Moderately worried | 2,838 (14.7) |
| 3 Not too worried | 5,351 (26.5) |
| 4 Not worried at all | 9,850 (50.0) |
| How worried are you about paying monthly bills, n (weighted %) |  |
| 1 Very worried | 1,559 (7.9) |
| 2 Moderately worried | 3,526 (17.7) |
| 3 Not too worried | 5,380 (26.3) |
| 4 Not worried at all | 9,356 (48.1) |
| How worried are you about paying rent/mortgage/housing costs, n (weighted %) | |
| 1 Very worried | 1,318 (6.7) |
| 2 Moderately worried | 2,559 (12.3) |
| 3 Not too worried | 5,082 (23.4) |
| 4 Not worried at all | 10,862 (57.6) |
